# Association Between Serum Afamin Levels with Nonalcoholic Associated Fatty Liver Disease

**DOI:** 10.1155/2022/7175108

**Published:** 2022-06-28

**Authors:** Shenghui Chen, Zhening Liu, Li Cen, Jinghua Wang, Juanwen Zhang, Xiaofeng Zhang, Chengfu Xu

**Affiliations:** ^1^Gastroenterology, Affiliated Hangzhou First People's Hospital, Zhejiang University School of Medicine, Hangzhou 310006, Zhejiang, China; ^2^Gastroenterology, The First Affiliated Hospital, Zhejiang University School of Medicine, Hangzhou 310003, Zhejiang, China; ^3^Hangzhou Hospital & Institute of Digestive Diseases, Hangzhou 310006, Zhejiang, China; ^4^Key Laboratory of Integrated Traditional Chinese and Western Medicine for Biliary and Pancreatic Diseases of Zhejiang Province, Hangzhou 310006, Zhejiang, China; ^**5**^ Department of Laboratory Medicine, the First Affiliated Hospital, Zhejiang University School of Medicine, Hangzhou 310003, Zhejiang, China

## Abstract

Afamin is a member of the hepatokine that are strongly associated with various metabolic diseases. The relationship between afamin and nonalcoholic fatty liver disease (NAFLD) remains unclear. This study aimed to explore the correlation between serum afamin levels and NAFLD. We analyzed 88 NAFLD patients and 88 age- and sex-matched healthy controls who took their health examinations at the First Affiliated Hospital, Zhejiang University School of Medicine. The association was further confirmed in 22 biopsy-confirmed NAFLD patients and 36 healthy controls. Serum afamin levels were evaluated using an enzyme-linked immunosorbent assay (ELISA). NAFLD patients had significantly higher serum afamin levels than the healthy controls (14.79 ± 5.04 mg/L versus 10.83 ± 3.24 mg/L; *P* < 0.001). Serum afamin levels were positively correlated with metabolic parameters including the body mass index, waist circumference, systolic blood pressure, liver enzymes, and lipid profiles. A multiple regression analysis showed that serum afamin levels were independently related to the risk of NAFLD (OR: 1.289, 95% CI, 1.141–1.456; *P* < 0.001). The receiver operating characteristic (ROC) analysis showed that the area under curve (AUC) of serum afamin plus the BMI for detecting NAFLD was 0.878. In participants with liver biopsies, the serum afamin plus the BMI detected NAFLD with an AUC of 0.758. In conclusion, serum afamin levels were positively associated with prevalence and risk of NAFLD, and serum afamin plus the BMI had a high diagnostic performance for NAFLD. This study provides epidemiological evidence of afamin in NAFLD.

## 1. Introduction

Nonalcoholic fatty liver disease (NAFLD) has become one of the most common chronic liver diseases, affecting approximately 25% adults worldwide [1]. The prevalence of NAFLD in Asia has rapidly climbed to approximately 29.62% in recent years [2]. The increased NAFLD prevalence is strongly associated with the increasing incidence of obesity and related metabolic disorders, such as type 2 diabetes mellitus, chronic kidney disease, and cardiovascular diseases, and is considered a major public health concern and socioeconomic burden [3–5].

The liver is a crucial regulator of systemic metabolism and energy homeostasis [6]. Hepatokines, known as liver-enriched secreted proteins, are the proteins or protein-like substances that are primarily secreted by the liver [7]. An increasing number of studies found that hepatokines play significant regulatory roles in lipid and glucose metabolism [8, 9]. Clinical investigations have found that altered hepatokines were significantly associated with metabolic syndrome (MetS) and cardiovascular diseases [7, 8, 10]. Similar to the function of adipokines and myokines, hepatokines play a significant role in the development of fatty liver disease. For example, FGF21, which is primarily secreted by the liver, modulates the progress of NAFLD by regulating oxidation stress, endoplasmic reticulum stress, mitochondria dysfunction, and inflammation [11].

Afamin is the fourth member of the albumin gene family and is primarily expressed and secreted in the liver [12]. Although the physiologic function of afamin is largely unknown, current investigations have found that afamin is closely correlated with obesity and related metabolic diseases [9]. Serum afamin levels have been found to be positively associated with the body mass index (BMI) [13] and the prevalence of MetS and its components [14]. Serum afamin levels were also significantly associated with type 2 diabetes mellitus [15]. In addition, transgenic mice with an overexpression of afamin are characterized by elevated body weight and serum levels of lipids and lipoproteins [14]. Furthermore, NAFLD is also characterized with increased inflammatory burden [16]. And recent studies have reported that afamin was associated with inflammatory conditions, such as polycystic ovary syndrome [17]. However, whether serum afamin levels are associated with NAFLD, a hepatic manifestation of MetS, remains unclear.

In this study, we explored the association between serum afamin levels and NAFLD. We also explored whether serum afamin could serve as a potential biomarker for the noninvasive diagnosis of NAFLD.

## 2. Materials and Methods

### 2.1. Study Participants

A total of 88 NAFLD patients and 88 age- and gender-matched healthy controls were recruited from individuals who took their health examination at the First Affiliated Hospital, Zhejiang University School of Medicine between September 25 and October 10, 2020. Participants were excluded as follows: (i) those with alcohol consumption ≥ 210 g/week for men or ≥ 140 g/week for women; (ii) those with a history of viral hepatitis or other chronic liver disease; (iii) those taking antidiabetic, antihypertension, or lipid-lowering drugs; and (iv) those with acute infections within 2 weeks or with a history of malignancy. To validate the diagnostic value of afamin on NAFLD, another 22 biopsy-confirmed NAFLD patients and 36 healthy controls were enrolled from the First Affiliated Hospital, Zhejiang University School of Medicine. All of the liver biopsies were performed due to suspected NAFLD during liver surgeries. This study was approved by the Ethics Committee of the First Affiliated Hospital, Zhejiang University School of Medicine (reference number: 2021-007).

### 2.2. Clinical and Biochemical Measurements

Data on the demographic characteristics of the participants were collected using a self-administered health questionnaire. Detailed physical measurements such as weight, height, waist circumference, and systolic and diastolic blood pressure were further measured during the health examination [18]. The BMI was calculated using the kilograms of weight divided by the square of the height in meters.

Serum blood samples of all the participants for the biochemical analysis of metabolic parameters were obtained using venipuncture after an overnight fast. Serum biochemical parameters such as alanine aminotransferase (ALT), aspartate aminotransferase (AST), *γ*-glutamyl transpeptidase (GGT), uric acid, triglyceride (TG), total cholesterol (TC), low-density lipoprotein (LDL)-cholesterol and high-density lipoprotein (HDL)-cholesterol, and fasting blood glucose (FBG) were measured using an automatic biochemistry analyzer (Olympus, Kobe, Japan) according to the manufacturer's instructions. Hemoglobin, platelets, and white blood cell counts were determined using a SYSMEX XT-1800 hematology autoanalyzer with standard methods.

During the health examinations, an abdominal ultrasound examination was performed by trained ultrasonographists using a Sonoline G60S ultrasound system with a 3.5-MHz probe (Siemens, Erlangen, Germany) [19].

### 2.3. Afamin Measurement

The serum afamin concentration was measured in all included participants using an ELISA (ABIN415271, Fernhurst, TX) according to the manufacturer's instructions. The maximum detectable dose of human afamin was determined to be 0.2 mg/L. Serum blood samples were diluted 1:1000 with phosphate buffering solution (PBS) depending on the available concentration of the ELISA kit, and of all diluted samples were in the detection range of the ELISA kit.

### 2.4. Diagnostic Criteria and Definitions

NAFLD was defined as the presence of fatty liver with the absence of excessive alcohol use or other identifiable causes. Fatty liver was diagnosed using an abdominal ultrasound examination, and the criteria were based on those suggested by the Chinese Liver Disease Association [20]. Dyslipidemia was defined according to the Chinese guidelines on prevention and treatment of dyslipidemia in adults [21]. Hypertension was defined as systolic blood pressure ≥ 140 mm Hg or diastolic blood pressure ≥90 mm Hg [22]. According to the WHO, an adult with a BMI ≥ 25 kg/m^2^ was considered overweight. Type 2 diabetes mellitus was defined as a fasting glucose level ≥ 126 mg/dL (≥7 mmol/L) according to the 1997 American Diabetes Association criteria [23].

### 2.5. Statistical Analyses

Statistical analyses were performed using the SPSS software package version 25.0 for Mac (SPSS Inc., Chicago, IL). First, normality analysis was conducted for continuous variables by Kolmogorov–Smirnov test. Categorical variables were expressed as frequencies with percentages, and continuous variables were presented as means ± standard deviations (SD). Student's *t*-test or one-way ANOVA was conducted for continuous variables, while chi-square tests were conducted for the categorical variables. The correlation between the serum afamin levels and metabolic parameters was examined using a Spearman correlation analysis. The Cochran–Armitage trend test was used to show the trend of the prevalence. Serum afamin levels were further divided into quartiles to conduct a quartile-based analysis. Multivariable logistic regression was further used to analyze the serum afamin levels and related factors associated with the likelihood of NAFLD (probability to enter= 0.05 and probability to remove= 0.10). In order to generate the predicted probability of NAFLD, we included the BMI and afamin, separately and simultaneously, in the logistic regression model. Then, the receiver operating characteristic (ROC) curve was used to evaluate the diagnostic performance of serum afamin levels for NAFLD. The criterion for selecting the optimum cutoff point depended on the Youden's index. A *P* value < 0.05 (two-tailed) was considered to be statistically significant.

## 3. Results

### 3.1. Serum Afamin Levels Were Elevated in NAFLD Patients

A total of 88 NAFLD patients and 88 age- and gender-matched healthy controls were enrolled for measuring the serum afamin levels.  Table 1 illustrates the clinical characteristic comparisons between the NAFLD patients and the healthy controls. The mean age was 47.64 ± 13.94 years old, and females comprised 36.4% of the NAFLD patients. Compared with healthy controls, NAFLD patients had higher BMI, waist circumference, higher serum levels of ALT, GGT, TG, and uric acid, and lower serum HDL-cholesterol levels (Table 1). Interestingly, we found that the NAFLD patients had significantly higher serum afamin concentrations than the healthy controls (14.79 ± 5.04 mg/L versus 10.83 ± 3.24 mg/L, *P* < 0.001; Table 1). This finding suggests that elevated serum afamin levels may be correlated with NAFLD.

### 3.2. Serum Afamin Levels Were Positively Associated with Metabolic Parameters

The Spearman correlation analysis is shown in Table 2. Serum afamin levels were positively correlated with the BMI (*r*_*s*_ = 0.242, *P* = 0.001), waist circumference (*r*_*s*_ = 0.207, *P* = 0.010), and systolic blood pressure (*r*_*s*_ = 0.203, *P* = 0.007). We also found that the serum afamin levels were positively correlated with liver enzymes and lipid profiles, including ALT (*r*_*s*_ = 0.207, *P* = 0.006), GGT (*r*_*s*_ = 0.247, *P* = 0.001), and TG (*r*_*s*_ = 0.189, *P* = 0.012). All of the participants were further divided into quartiles according to their afamin levels, and it was found that participants with the highest afamin quartile had the higher GGT and diastolic blood pressure than those with the lowest quartile (Table S1). These results suggest a positive association between serum afamin levels and metabolic parameters.

### 3.3. Serum Afamin Levels Were Positively Associated with Prevalence of NAFLD

**Figure 1:**
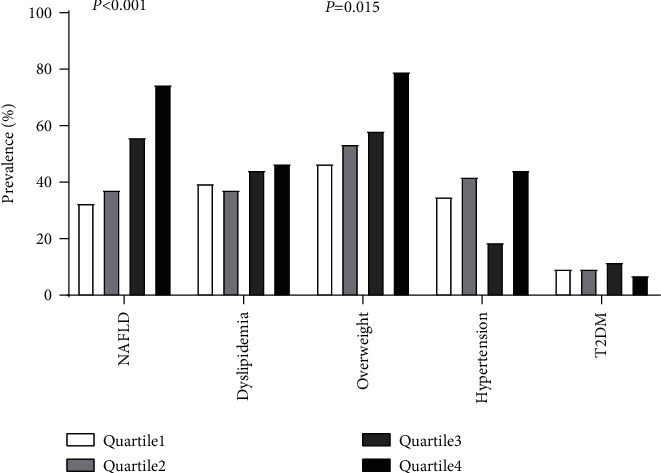
Prevalence of NAFLD, dyslipidemia, overweight, hypertension, and T2DM according to quartiles of the serum afamin levels. Participants were classified into quartiles according to their serum afamin levels. Quartile 1 < 9.78 mg/L, 9.78 ≤ Quartile 2 < 12.14 mg/L, 12.14 ≤ Quartile 3 < 15.29 mg/L, and Quartile 4 ≥ 15.29 mg/L. NAFLD= nonalcoholic fatty liver disease; T2DM = type 2 diabetes mellitus. P-trend was calculated by the Cochran–Armitage *χ*2 test for the trend.

Of the 176 participants enrolled, 75 fulfilled the diagnostic criteria of dyslipidemia, 105 were overweight, 16 had type 2 diabetes mellitus, and 59 had hypertension. As shown in Figure 1, serum afamin quartiles were positively associated with the prevalence of NAFLD (*P* < 0.001) and were overweight (P = 0.002), while there was no significant association between the serum afamin quartiles and the prevalence of T2DM, dyslipidemia, or hypertension. These results further suggested a positive association between the serum afamin levels with NAFLD.

Elevated serum afamin levels were associated with an increased risk of NAFLD.

Univariable and multivariable logistic regression analyses were then performed to explore the association of serum afamin levels with the risk of NAFLD (Table 3). In the univariable analysis, we found that elevated serum afamin levels were significantly associated with an increased risk of NAFLD (OR: 1.287, 95% CI: 1.167–1.419; *P* < 0.001). In the multivariable analysis, serum afamin levels remained significantly and positively associated with the risk of NAFLD (OR: 1.289, 95% CI, 1.141–1.456; *P* < 0.001). These results suggested that the participants with elevated afamin levels may have an increased risk of NAFLD.

### 3.4. Diagnostic Performance of Serum Afamin in NAFLD

**Figure 2:**
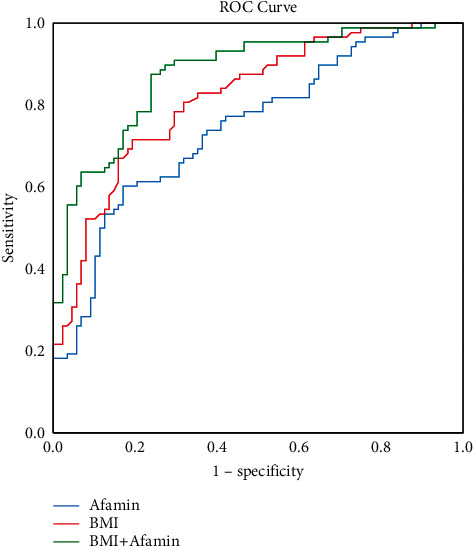
Receiver operating characteristic (ROC) analysis for the diagnosis of NAFLD. The ROC curves for afamin, BMI, and afamin plus the BMI are shown. The area under the curve (AUC) of afamin was 0.746, and the positive predictive value was 62.5%. The negative predictive value was 69.3%, and the accuracy was 65.9%. The area under the curve (AUC) of the BMI was 0.818, and the positive predictive value was 77.3%. The negative predictive value was 70.5%, and the accuracy was 73.9%. The area under the curve (AUC) of afamin plus the BMI was 0.878, and the positive predictive value was 77.3%. The negative predictive value was 79.5%, and the accuracy was 78.4%.

The area under the ROC curves was calculated to measure the sensitivity and specificity of the variables with statistical significance, as shown in Table 3, as a potential marker for NAFLD. We found that the calculated AUC value was 0.746 for afamin, 0.818 for the BMI, and 0.878 for afamin plus the BMI (Figure 2). The cutoff for detecting NAFLD based on the distribution of specificities and sensitivities was also calculated. For afamin, a cutoff value of ≥ 13.50 mg/L with a sensitivity of 60.2% and a specificity of 83.0% was used to detect NAFLD. Using this threshold, the positive predictive value was 62.5%, the negative predictive value was 69.3%, and the accuracy was 65.9%. For the serum afamin levels plus BMI, the positive predictive value was 77.3%, the negative predictive value was 79.5%, and the accuracy was 78.4%, indicating a better diagnostic potential than that of the BMI or the serum afamin levels alone.

### 3.5. Validation of Serum Afamin in Liver Biopsy-Proved Participants

**Figure 3:**
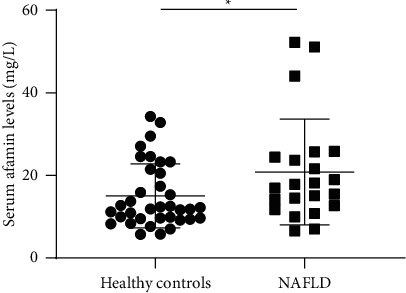
Differentially serum afamin levels in biopsy-proven participants. A total of 58 biopsy-proved participants including 22 biopsy-confirmed NAFLD patients and 36 healthy controls were enrolled. The serum afamin levels were significantly higher in the biopsy-proved NAFLD patients than in the healthy controls (*P* < 0.01). A significance analysis was conducted using Student's *t*-test.

**Figure 4:**
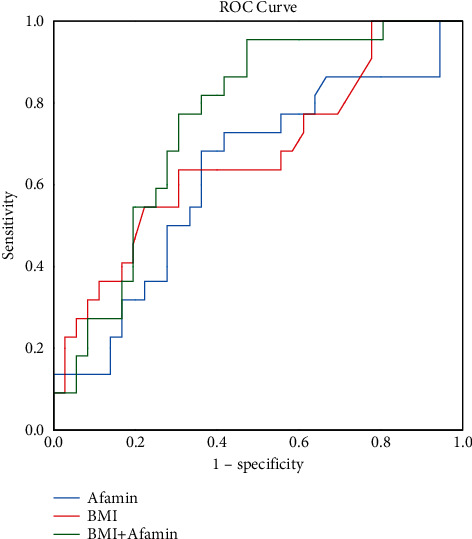
The receiver operating characteristic (ROC) analysis of afamin, body mass index (BMI), and BMI plus afamin for detecting NAFLD in biopsy-proven participants. The ROC curves for afamin, the BMI, and afamin plus the BMI are shown. The area under the curve (AUC) of afamin was 0.628. The area under the curve (AUC) of the BMI was 0.670. The area under the curve (AUC) of afamin plus the BMI was 0.758.

The diagnostic performance of serum afamin in 58 liver biopsy-proved participants, including 22 NAFLD patients and 36 controls, was validated. We found that the biopsy-proved NAFLD patients had significantly higher serum afamin levels than the healthy controls (*P* < 0.01; Figure 3). As shown in Figure 4, the AUC value of the serum afamin, BMI, and afamin plus the BMI for detecting NAFLD was 0.628, 0.670, and 0.758, respectively, suggesting a good predictive value of serum afamin plus the BMI in the liver biopsy-proved participants.

## 4. Discussion

In this study, we presented evidence for the first time that serum afamin levels were significantly elevated in NAFLD patients, and the levels were positively associated with the prevalence and risk of NAFLD. Moreover, we found that serum afamin levels have high diagnostic performance for NAFLD diagnosis. These findings may have important clinical implications for the diagnosis and treatment of NAFLD.

Afamin is a hepatokine that is highly expressed in the liver and secreted into blood. Similar to the function of other hepatokines such as FGF21, SMOC1, LECT2, and SeP, afamin plays a significant role in lipid and glucose metabolism [24–27]. Afamin acts as a transfer protein that is responsible for exchanging lipoproteins, such as cholesterol, triglycerides, and apoB [28]. The overexpression of afamin significantly increases the levels of serum triglycerides, total cholesterol, and FBG [14]. However, the association between the serum afamin levels and NAFLD has not been explored as yet. In this study, our findings suggested that afamin may be also closely associated with NAFLD.

Afamin has been previously reported to be a potential biomarker for gestational diabetes mellitus [29], preeclampsia [30], and type 2 diabetes mellitus [15]. Consistent with these reports, it was found in this study that the serum afamin levels had a good diagnostic value for NAFLD. At a cutoff value of ≥ 13.50 mg/L, the diagnostic accuracy of afamin was calculated to be 65.9%, with a specificity of 83.0%, to detect NAFLD. In addition, the diagnostic accuracy of afamin plus the BMI turned out to be as high as 78.4% (AUC = 0.878), with a specificity of 79.5%. The diagnostic performance of afamin plus the BMI was also verified in biopsy-proven participants, and it was found that the BMI plus afamin had a high accuracy for the diagnosis of NAFLD. These findings suggested that afamin may serve as a potential biomarker for the noninvasive diagnosis of NAFLD. In recent years, several pharmacological therapies aiming to alleviate NAFLD are being examined at various phases of clinical trials; hence, the early diagnosis of NAFLD is useful to try to cure this very common disease [31].

The mechanistic explanation for the association between afamin and NAFLD remains unclear. Afamin has been shown to be a specific binding protein for vitamin E and may be responsible for the transport of vitamin E [28]. Vitamin E is identified as a potent antioxidant that has anti-inflammatory effects and has been used in the treatment of fatty liver diseases. A systematic review and meta-analysis have revealed that adjuvate vitamin E therapy significantly improved biochemical and histological parameters in fatty liver patients [32]. Vitamin E therapy reduced the levels of markers of oxidative stress and the histological fibrosis score in the livers of MCD diet-induced models of steatohepatitis mice [33]. Afamin has also been reported to bind tightly to Wnt proteins and acylated Wnt proteins in active form. The Wnt signaling pathway is ubiquitous signaling that regulates a wide range of physiologic processes in various diseases, such as MetS and NAFLD [34, 35]. The knockout of LRP6, a receptor for canonical Wnt ligands, was shown to significantly induce liver steatosis through active hepatic AKT/PI3K/mTOR [36]. Previous studies have also reported that elevated serum afamin levels are associated with insulin resistance and with increased glucose concentrations [13, 14], which are involved in the development of NAFLD. Therefore, afamin may play a role in the progress of NAFLD through regulated vitamin E metabolism, insulin resistance, and affect the Wnt-dependent mechanism. However, further studies are required to confirm this hypothesis.

There are several limitations in this study. First, this is an observational design, and further studies are required to explore the potential causal relationship between serum afamin levels in the development of NAFLD. Second, a relatively limited sample size was recruited from a single center in this study. Further studies with a larger sample size may be needed to confirm the association between serum afamin and NAFLD, as well as to verify the accuracy of afamin in the diagnosis of NAFLD.

## 5. Conclusion

In conclusion, this study revealed that the serum afamin levels were positively associated with prevalence and risk of NAFLD. These results also suggested that serum afamin may be a reliable biomarker for the noninvasive diagnosis of NAFLD.

## Figures and Tables

**Table 1:** Clinical characteristics of NAFLD patients and healthy controls.

Variables	Healthy controls (*n* = 88)	NAFLD patients (*n* = 88)	*P* value
Age (years)	47.85 ± 12.86	47.64 ± 13.94	0.804^a^
Gender (female/male)	32/56	32/56	1.000^b^
Body mass index (kg/m^2^)	23.04 ± 2.22	26.3 ± 2.53	<0.001^a^
Waist circumference (cm)	80.03 ± 6.15	87.37 ± 12.31	<0.001^a^
Systolic blood pressure (mm Hg)	123.2 ± 18.75	128.68 ± 24.56	0.098^a^
Diastolic blood pressure (mm Hg)	74.06 ± 11.99	77.43 ± 11.92	0.063^a^
Alanine aminotransferase (U/L)	22.61 ± 21.91	42.65 ± 57.38	0.003^a^
Aspartate aminotransferase (U/L)	21.06 ± 11.4	28.7 ± 35.77	0.058^a^
*γ*-Glutamyl transpeptidase (U/L)	29.92 ± 26.39	44.97 ± 36.91	0.002^a^
Uric acid (*μ*mol/L)	331.24 ± 71.39	373.6 ± 89.62	0.001^a^
Triglyceride (mmol/L)	1.43 ± 1.06	2.27 ± 1.75	<0.001^a^
Total cholesterol (mmol/L)	4.70 ± 0.92	4.89 ± 1.02	0.201^a^
High-density lipoprotein cholesterol (mmol/L)	1.27 ± 0.30	1.12 ± 0.27	<0.001^a^
Low-density lipoprotein cholesterol (mmol/L)	2.73 ± 0.78	2.83 ± 0.78	0.441^a^
Fast blood glucose (mmol/L)	5.25 ± 1.51	5.43 ± 1.32	0.415^a^
Afamin (mg/L)	10.83 ± 3.24	14.79 ± 5.04	<0.001^a^

Data are expressed as the means ± standard deviations. ^a^ Student's *t*-test.^ b^ Pearson chi-square test.

**Table 2:** Correlation of the serum afamin levels with the anthropometric and biochemical variables.

Variables	*r *_*s*_	*P* value
Age (years)	0.063	0.408
Body mass index (kg/m^2^)	0.242	0.001
Waist circumference (cm)	0.207	0.010
Diastolic blood pressure (mm Hg)	0.147	0.052
Systolic blood pressure (mm Hg)	0.203	0.007
Alanine aminotransferase (U/L)	0.207	0.006
Aspartate aminotransferase (U/L)	0.154	0.041
*γ*-Glutamyl transpeptidase (U/L)	0.247	0.001
Uric acid (*μ*mol/L)	0.145	0.055
Triglyceride (mmol/L)	0.189	0.012
Total cholesterol (mmol/L)	0.050	0.511
High-density lipoprotein cholesterol (mmol/L)	−0.054	0.475
Low-density lipoprotein cholesterol (mmol/L)	−0.035	0.642
Fast blood glucose (mmol/L)	0.145	0.055

The significance analysis was conducted using the Spearman rank correlation test.

**Table 3:** Logistic regression analysis of NAFLD with the anthropometric and biochemical variables.

Variables	Univariable analysis	Multivariable analysis^*∗*^
	OR (95%CI)	OR (95%CI)
Age (years)	0.997 (0.975–1.020)	
Body mass index (kg/m^2^)	1.875 (1.539–2.285)	1.794 (1.458–2.208)
Waist circumference (cm)	1.129 (1.072–1.189)	
Diastolic blood pressure (mm Hg)	1.012 (0.997–1.027)	
Systolic blood pressure (mm Hg)	1.024 (0.998–1.051)	
Alanine aminotransferase (U/L)	1.029 (1.010–1.048)	
Aspartate aminotransferase (U/L)	1.030 (0.997–1.063)	
*γ*-Glutamyl transpeptidase (U/L)	1.016 (1.005–1.028)	
Uric acid (*μ*mol/L)	1.007 (1.003–1.011)	
Triglyceride (mmol/L)	2.181 (1.464–3.250)	1.627 (1.032–2.566)
Total cholesterol (mmol/L)	1.224 (0.898–1.669)	
High-density lipoprotein cholesterol (mmol/L)	0.148 (0.048–0.456)	
Low-density lipoprotein cholesterol (mmol/L)	1.163 (0.793–1.707)	
Fast blood glucose (mmol/L)	1.094 (0.881–1.358)	
Afamin (mg/L)	1.287 (1.167–1.419)	1.289 (1.141–1.456)

^*∗*^Backward stepwise regression was used in the multivariate logistic regression analyses (probability to enter= 0.05, and probability to remove = 0.10). All variables in the table entered the regression model at first. Finally, the body mass index, triglyceride, and afamin were retained in the multivariable model. OR= odds ratio, CI= confidence interval.

## Data Availability

The data used to support the findings of this study are available from the corresponding author upon request.

## Supplementary Materials

Supplementary Materials
*Table S1.* Clinical characteristics by quartiles of the serum afamin levels. See table S1 in the Supplementary Material.
