# Supplementary material for: Association Between Serum Afamin Levels with Nonalcoholic Associated Fatty Liver Disease
Source: Can J Gastroenterol Hepatol. 2022 Jun 28;2022:7175108. doi: 10.1155/2022/7175108 (PMC9256457; doi:10.1155/2022/7175108)
Supplement: Supplementary Materials — Table S1. Clinical characteristics by quartiles of the serum afamin levels. See table S1 in the Supplementary Material. [file 7175108.f1.docx]

**Table S1. Clinical characteristics by quartiles of the serum afamin levels.**

| Variables | Quartile 1 (*n*=44) | Quartile 2 (*n*=44) | Quartile 3 (*n*=44) | Quartile 4 (*n*=44) | *P* value |
| --- | --- | --- | --- | --- | --- |
| Afamin (mg/L) | 7.84±1.35 | 10.75±0.68 | 13.79±0.97 | 18.88±4.12 | <.0001 |
| Age (year) | 46.73±12.10 | 48.25±14.15 | 46.93±14.51 | 49.07±12.92 | 0.825 |
| Body mass index (kg/m²) | 24.14±2.83 | 24.37±3.30 | 24.52±2.60 | 25.65±2.62 | 0.065 |
| Waist circumference (cm) | 81.02±15.42 | 83.66±8.22 | 83.54±7.22 | 86.48±7.49 | 0.124 |
| Systolic blood pressure (mmHg) | 119.75±16.12 | 131.27±17.93 | 123.93±16.51 | 128.82±32.06 | 0.064 |
| Diastolic blood pressure (mmHg) | 71.70±12.16 | 77.25±11.50 | 75.11±9.99 | 78.91±13.44 | 0.031 |
| Alanine aminotransferase (U/L) | 25.09±19.07 | 30.36±30.08 | 31.20±49.49 | 43.86±64.28 | 0.238 |
| Aspartate aminotransferase (U/L) | 20.70±6.48 | 23.80±15.56 | 22.70±17.16 | 32.32±47.45 | 0.184 |
| γ-Glutamyl transpeptidase (U/L) | 35.36±31.92 | 29.86±23.98 | 35.05±25.85 | 49.50±43.79 | 0.033 |
| Uric acid (μmol/L) | 337.25±73.16 | 341.86±81.21 | 364.77±93.99 | 365.8±83.24 | 0.237 |
| Triglyceride (mmol/L) | 1.61±1.11 | 1.80±2.14 | 1.93±1.30 | 2.05±1.24 | 0.549 |
| Total cholesterol (mmol/L) | 4.84±0.87 | 4.75±1.16 | 4.74±0.90 | 4.85±0.98 | 0.937 |
| High-density lipoprotein cholesterol (mmol/L) | 1.24±0.30 | 1.19±0.30 | 1.17±0.28 | 1.17±0.30 | 0.636 |
| Low-density lipoprotein cholesterol (mmol/L) | 2.87±0.74 | 2.77±1.01 | 2.73±0.66 | 2.75±0.68 | 0.861 |
| Fast blood glucose (mmol/L) | 5.1±0.98 | 5.48±1.64 | 5.5±1.61 | 5.28±1.34 | 0.520 |

Data are expressed as the means ± standard deviations. The participants were classified into quartiles according to their serum afamin levels. Quartile 1 <9.78 mg/L, 9.78≤Quartile 2 <12.14 mg/L, 12.14 ≤Quartile 3 <15.29 mg/L, and Quartile 4 ≥15.29 mg/L. A significance analysis was conducted using a one-way ANOVA test.
